# Supplementary material for: TDP-43 pathology triggers neuroinflammation and cognitive impairment by inducing microglial necroptosis
Source: EMBO Mol Med. 2026 Mar 10;18(4):1318–41. doi: 10.1038/s44321-026-00394-9 (PMC13083925; doi:10.1038/s44321-026-00394-9)
Supplement: Supplementary file 3 — Appendix [file 44321_2026_394_MOESM3_ESM.pdf]

Appendix

Title: TDP-43 pathology triggers neuroinflammation and cognitive impairment by inducing microglial necroptosis

Authors' information: Shenrui Guo, Hongfu Jin, Hui Sun, Shuo Huang, Yuanyuan Chen, Yuge Chang, Yu Zhang, Lin Ding, Suyun Chen, Chenglai Fu, Yafu Yin, Weiwei Cheng

Appendix Figure S1	Page 2
Appendix Figure S2	Page 3
Appendix Figure S3	Page 4

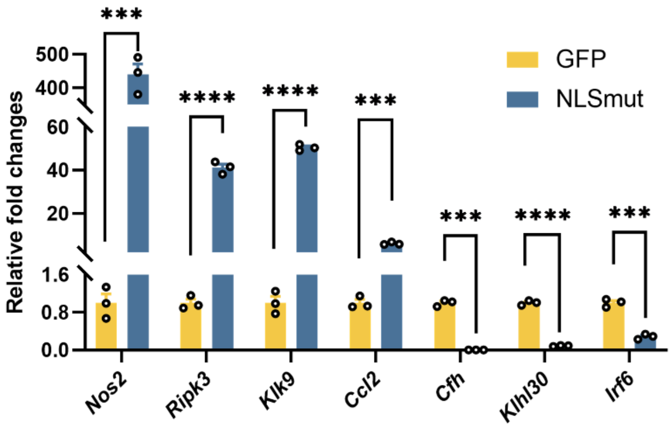

Appendix Fig.S1 (relative to Fig.2) qRT-PCR analysis validated the positive hits from RNAseq. Data are mean ± SEM from 3 biological replicates and analyzed by unpaired two-tailed Student's *t* test. For *Nos*, p=0.00016; for *Ripk3*, p=0.00002; for *Klk9*, p=0.0000005; for *Ccl2*, p=0.00017; for *Cfh*, p=0.00026; for *Klhl30*, p=0.000007; for *Irf6*, p=0.00027. ***p < 0.001, ****p < 0.0001.

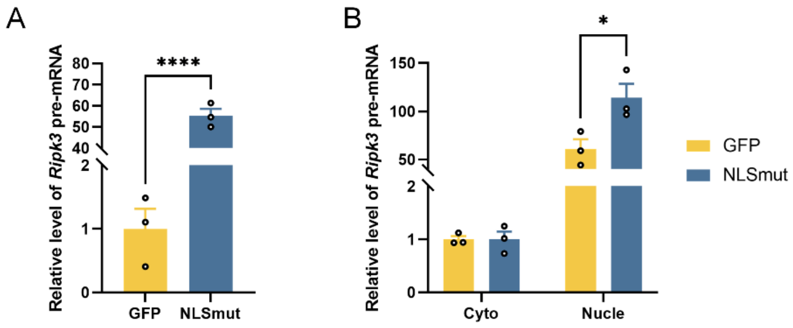

Appendix Fig.S2 (relative to Fig.5) *Ripk3* pre-mRNA was significantly elevated in BV2-NLSmut cells compared to BV2-GFP control cells. A Relative level of *Ripk3* pre-mRNA measured by qRT-PCR in BV2-GFP and BV2-NLSmut cells. Data are mean ± SEM from 3 biological replicates and analyzed by unpaired two-tailed Student's *t* test. p=0.00008. **B** Relative level of *Ripk3* pre-mRNA measured by qRT-PCR in either cytoplasmic or nucleus fraction (p=0.0397) from BV2-GFP and BV2-NLSmut cells. Data are mean ± SEM from 3 biological replicates and analyzed by unpaired two-tailed Student's *t* test. *$p < 0.05$, ****$p < 0.0001$.

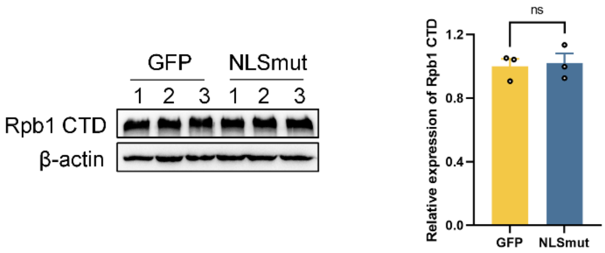

Appendix Fig.S3 (relative to Fig.5) Rpb1 expression level was comparable between BV2-NLSmut and BV2-GFP control cells. Immunoblotting and the corresponding quantification using antibody recognizing the C-terminal domain (CTD) of Rpb1 and β-actin in BV2-GFP and BV2-NLSmut cells. Data are mean ± SEM from 3 biological replicates and analyzed by unpaired two-tailed Student's *t* test. ns, not significant.
