# Supplementary material for: TDP-43 pathology triggers neuroinflammation and cognitive impairment by inducing microglial necroptosis
Source: EMBO Mol Med. 2026 Mar 10;18(4):1318–41. doi: 10.1038/s44321-026-00394-9 (PMC13083925; doi:10.1038/s44321-026-00394-9)
Supplement: Supplementary file 14 — Expanded View Figures [file 44321_2026_394_MOESM14_ESM.pdf]

Expanded View Figures

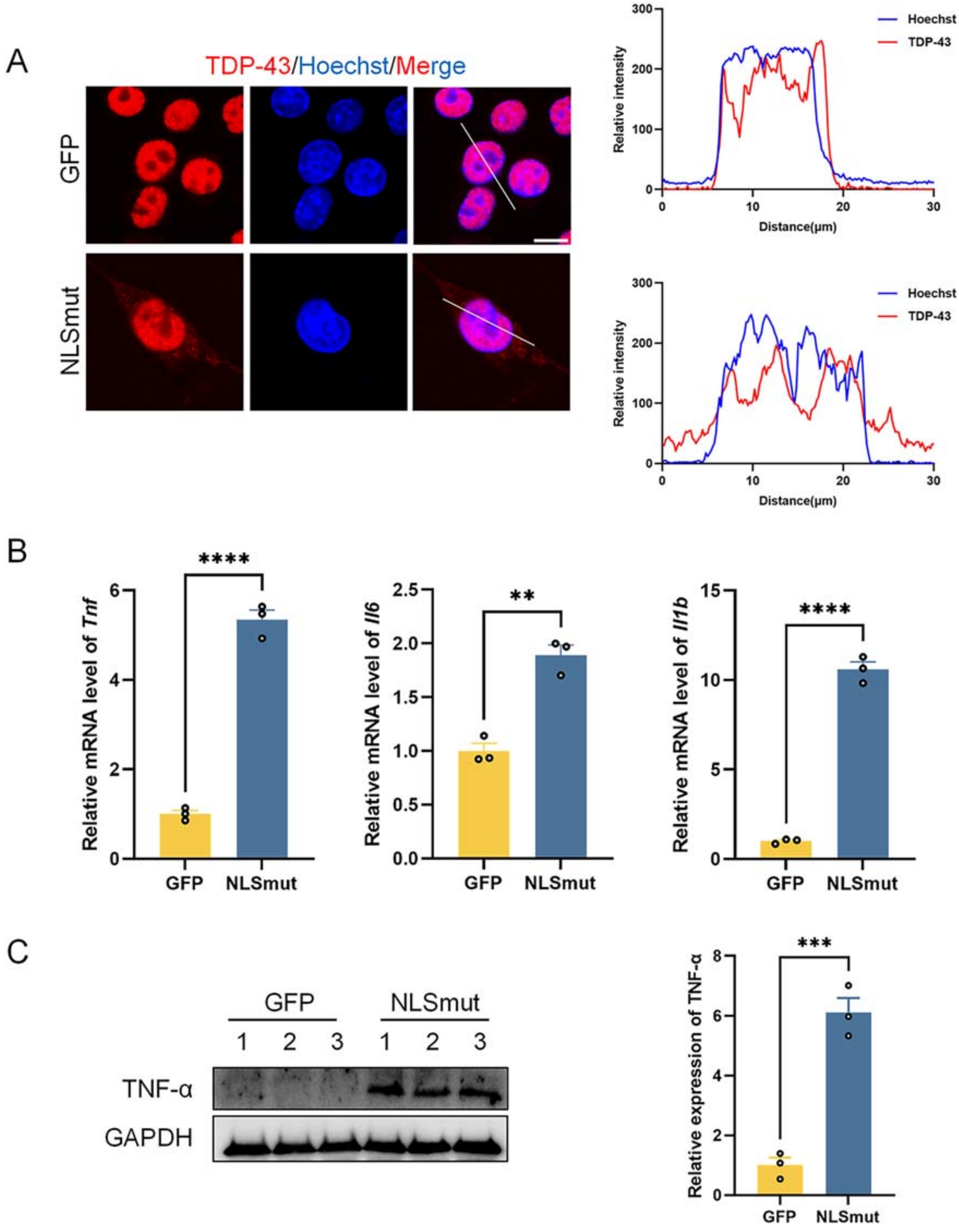

Figure EV1. (relative to Fig. 1) TDP-43 NLSmut induced neuroinflammation in BV2 microglial cells.

(**A**) Immunofluorescence and colocation analysis of TDP-43 (red) and Hoechst (blue) in BV2-GFP and BV2-NLSmut cells. Scale bar, 10 μm. (**B**) Relative mRNA level of *Tnf* ($p = 0.00005$), *Il6* ($p = 0.0016$) and *Il1b* ($p = 0.00002$) measured by qRT-PCR in BV2-GFP and BV2-NLSmut cells. Data are mean ± SEM from three biological replicates and analyzed by unpaired two-tailed Student's *t* test. (**C**) Immunoblotting using antibody recognizing TNF-α and GAPDH in BV2-GFP and BV2-NLSmut cells, along with the corresponding quantification. $p = 0.0008$. Data are mean ± SEM from 3 biological replicates and analyzed by unpaired two-tailed Student's *t* test. $^{**}p < 0.01$, $^{***}p < 0.001$, $^{****}p < 0.0001$.

© The Author(s)

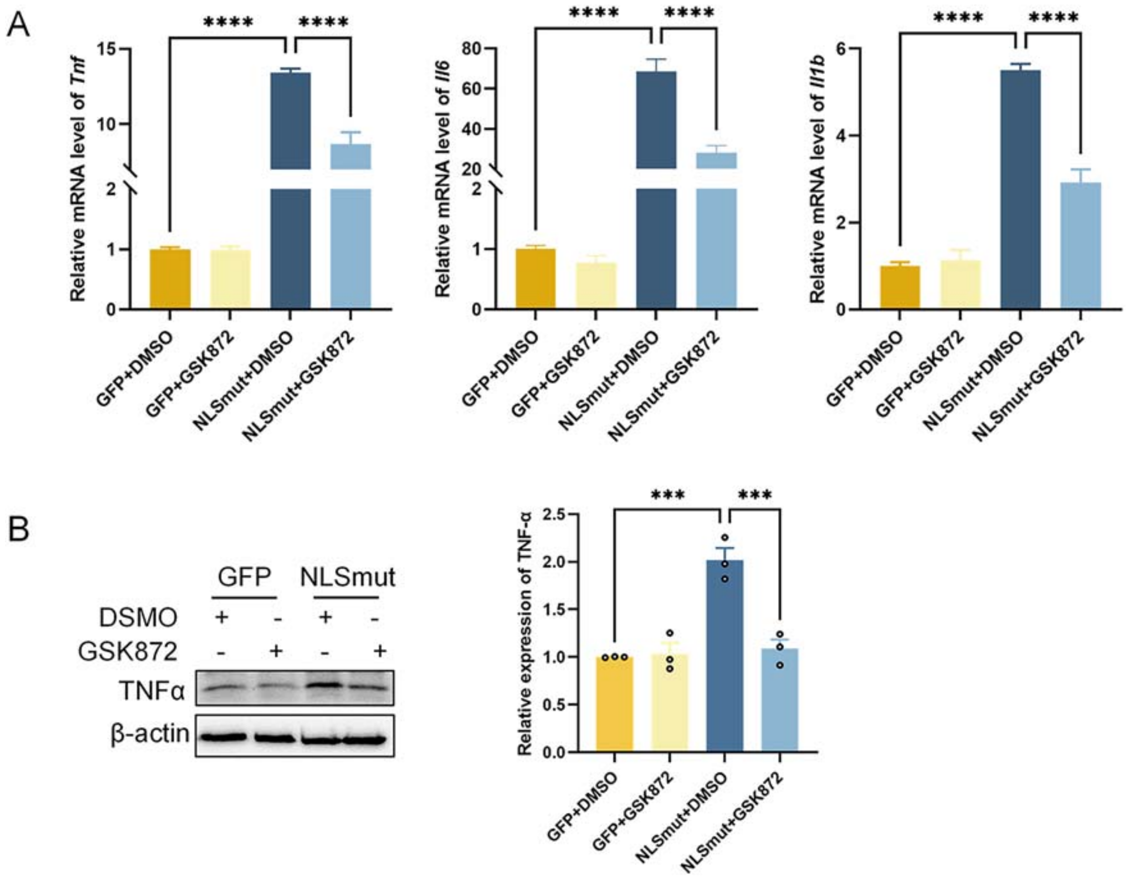

Figure EV2. (relative to Fig. 3) Pharmacologically inhibiting necroptosis suppressed TDP-43 NLSmut induced neuroinflammation.

(**A**) Relative mRNA level of *Tnf* (GFP + DMSO vs. NLSmut+DMSO, $p = 0.00000005$; NLSmut+DMSO vs. NLSmut+GSK872, $p = 0.00008$), *Il6* (GFP + DMSO vs. NLSmut +DMSO, $p = 0.000002$; NLSmut+DMSO vs. NLSmut+GSK872, $p = 0.00008$) and *Il1b* (GFP + DMSO vs. NLSmut+DMSO, $p = 0.0000006$; NLSmut+DMSO vs. NLSmut +GSK872, $p = 0.00004$) measured by qRT-PCR in BV2-GFP and BV2-NLSmut cells treated with or without GSK872 (5 µM, 24 h). Data are mean ± SEM from three biological replicates and analyzed by ANOVA followed by Bonferroni's multiple comparisons. (**B**) Immunoblotting using antibody recognizing TNF-α and β-actin in BV2-GFP and BV2-NLSmut cells treated with or without GSK872 (5 µM, 24 h), along with the corresponding quantification. GFP + DMSO vs. NLSmut+DMSO, $p = 0.0004$; NLSmut+DMSO vs. NLSmut+GSK872, $p = 0.0007$. Data are mean ± SEM from 3 biological replicates and analyzed by ANOVA followed by Bonferroni's multiple comparisons. ***$p < 0.001$, ****$p < 0.0001$.

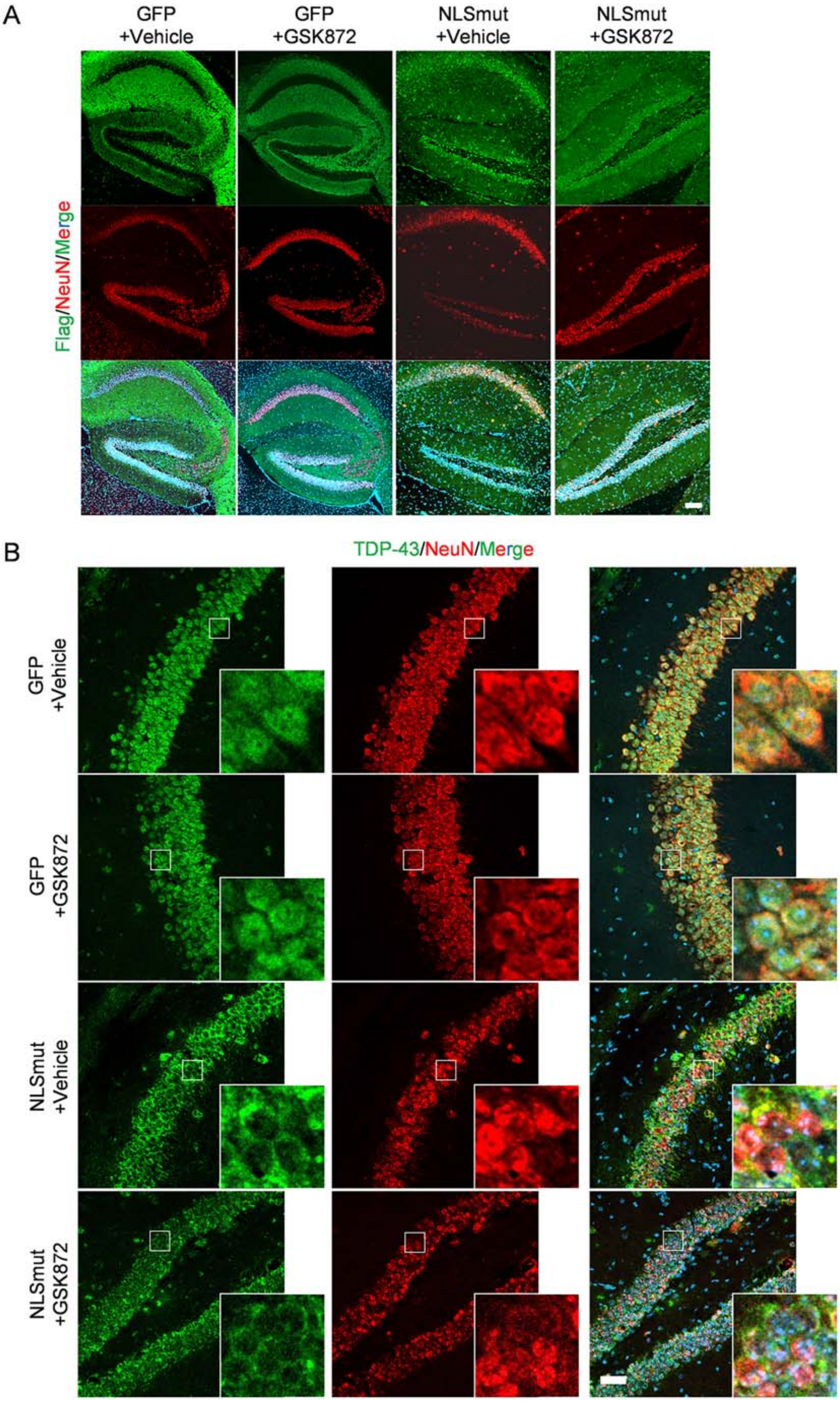
A
GFP +Vehicle
GFP +GSK872
NLSmut +Vehicle
NLSmut +GSK872
Flag/NeuN/Merge
B
TDP-43/NeuN/Merge
GFP +Vehicle
GFP +GSK872
NLSmut +Vehicle
NLSmut +GSK872

Figure EV3. (relative to Figs. 6 and 7) Immunofluorescence staining validated the overexpression of TDP-43 NLSmut in neurons in mice.

(**A**) Immunofluorescence of Flag (green) and NeuN (red) in GFP control and TDP-43-NLSmut mice treated with or without GSK872. Hoechst (blue) for nuclei. Scale bar, 100 μm. (**B**) Immunofluorescence of TDP-43 (green) and NeuN (red) in GFP control and TDP-43-NLSmut mice treated with or without GSK872. Hoechst (blue) for nuclei. Scale bar, 50 μm.

© The Author(s)

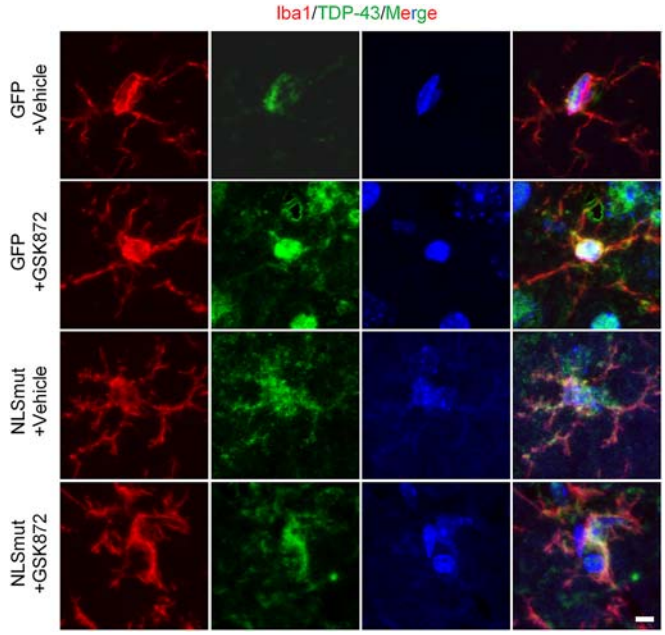

Figure EV4. (relative to Figs. 6 and 7) Immunofluorescence staining validated the overexpression of TDP-43 NLSmut in microglia in mice.

Immunofluorescence of TDP-43 (green) and Iba1 (red) in GFP control and TDP-43-NLSmut mice treated with or without GSK872. Hoechst (blue) for nuclei. Scale bar, 5 μm.

 © The Author(s)
